# Validation of Common Housekeeping Genes as Reference for qPCR Gene Expression Analysis During iPS Reprogramming Process

**DOI:** 10.1038/s41598-018-26707-8

**Published:** 2018-06-07

**Authors:** Yulia Panina, Arno Germond, Shinji Masui, Tomonobu M. Watanabe

**Affiliations:** 1 RIKEN Center for Biosystems Dynamics Research (BDR), 6-2-3 Furuedai, Suita, Osaka, 565-0874 Japan; 2 Graduate School of Frontier Biosciences, Osaka University, 1-3 Yamadaoka, Suita, Osaka, 565-0871 Japan; 3 Department of Life Science Frontiers, Center for iPS Cell Research and Application (CiRA), Kyoto University, 53 Kawahara-cho, Shogoin, Sakyo-ku, Kyoto, 606-8507 Japan

## Abstract

Induced pluripotent stem cell (iPS) reprogramming allows to turn a differentiated somatic cell into a pluripotent cell. This process is accompanied by many changes in fundamental cell properties, such as energy production, cell-to-cell interactions, cytoskeletal organization, and others. Real-time quantitative polymerase chain reaction (RT-qPCR) can be used as a quantitative method of gene expression analysis to investigate iPS reprogramming but it requires a validation of reference genes for the accurate assessment of target genes’ expression. Currently, studies evaluating the performance of reference genes during iPS reprogramming are lacking. In this study we analysed the stability of 12 housekeeping genes during 20 days of iPS reprogramming of murine cells based on statistical analyses of RT-qPCR data using five different statistical algorithms. This study reports strong variations in housekeeping gene stability during the reprogramming process. Most stable genes were Atp5f1, Pgk1 and Gapdh, while the least stable genes were Rps18, Hprt, Tbp and Actb. The results were validated by a proof-of-point qPCR experiment with pluripotent markers Nanog, Rex1 and Oct4 normalized to the best and the worst reference gene identified by the analyses. Overall, this study and its implications are particularly relevant to investigations on the cell-state and pluripotency in iPS reprogramming.

## Introduction

Induced pluripotent stem (iPS) cells were first developed by Shinya Yamanaka and his colleagues in 2006^1^, and are now the key technology for regenerative medicine and disease therapeutics. In addition, iPSCs were recently brought to attention as a model system for studying pluripotency mechanisms. A number of studies emerged that outlined key sequential stages involved in reprogramming process. The process is thought to progress from initiation, through maturation, to stabilization^2^. The earlier stages are associated with profound changes in cell behaviour such as mesenchymal-to-epithelial transition (MET)^3^, switch to glycolysis^4^ and changes in cell cycle progression patterns^5^, whereas later stages involve DNA demethylation^6,7^, telomerase activation and telomere elongation^8^ and activation of pluripotency gene network^9^.

Real-time quantitative polymerase chain reaction (RT-qPCR) is a powerful technique that allows to monitor relative changes in gene expression and is considered the “gold standard” in the field of mRNA quantification^10^. This technique requires a normalization strategy to ensure the reliability of the data^11,12^. One common strategy is to rely on the comparison of the target gene with an endogenous control (reference gene) in the same sample. At present, so-called housekeeping genes are universally used as a reference^13^. For example, housekeeping genes such as actin, ubiquitin or ribosomal genes are thought to be universally required for basic cellular functions and to be constitutively and stably expressed in varying physiological and experimental conditions. However, recent works have uncovered that housekeeping genes’ expression levels may vary depending on the gene, cell type and experimental conditions. For example, one of the most frequently used housekeeping genes, GAPDH (glyceraldehyde-3-phosphate dehydrogenase) has been found to be unstable depending on the type of tissue^14^, metabolic process^15^ or under certain experimental conditions^16^. Thus, confirming the stability of the normalizing gene of choice in cells under study is a prerequisite for a correct analysis of gene expression of any target gene.

To our best knowledge, the RT-qPCR analysis of common housekeeping genes’ stability over the time course of iPS reprogramming has never been performed. In this study, we conducted iPS reprogramming of a murine cell line and monitored changes in the expression of candidate housekeeping genes over time. The stability of housekeeping genes was assessed using five different statistical approaches described in previous literature. Our work has identified Atp5f1, Pgk1 and Gapdh as the most stably expressed genes, while Rps18, Hprt and Actb were found to fluctuate throughout the course of reprogramming. Using pluripotency markers Nanog, Rex1 and Oct4 whose levels are known to rise during reprogramming, we further demonstrated that choosing Rps18 as opposed to Atp5f1 would lead to inadequate normalization. Implications of our results are discussed in the context of iPS biology.

## Materials and Methods

### Cell culture and iPS reprogramming

iPS reprogramming was carried out in a reprogrammable cell system previously described by Hikichi and colleagues^17^. The system consists of a mouse neural progenitor cell line designated N31 which possesses three key characteristics of neural progenitors: (1) fibroblast growth factor and epidermal growth factor-dependent growth, (2) neural stem cell markers’ expression and (3) the ability to differentiate into neural lineages (described in detail in Han *et al*., 2012 and Hikichi *et al*., 2013). To bypass the need for mRNA or virus introduction into the cells, a doxycycline-inducible cassette with four Yamanaka factors, Oct4, Sox2, Klf4 and c-Myc, was permanently integrated into the cell genome^17^. Doxycycline addition results in the activation of the four factors and initiates reprogramming. Cells were seeded and kept on plastic gelatin-coated dishes in RHB neural stem cell media (#Y40000, Clontech Takara, Japan) supplemented with Ndiff (#Y40100, Clontech Takara, Japan) and 10 ng/ml FGF and 10 ng/ml EGF until they fully attached and spread. To initiate reprogramming, the medium was changed to Essential 8 iPS reprogramming medium (A1517001, Thermo Fisher, Japan) and 1 µg/ml doxycycline was added to the dish. From that point on, the media were changed every day to avoid pH fluctuations. The reprogramming was carried out until day 20, and the cell material samples (whole cell populations) were collected at 8 time points (on days 0, 1, 3, 5, 7, 10, 15, 20). One round of reprogramming thus yielded 8 cell pellets. The experiment (one full round of reprogramming) was repeated three times to obtain three biological replicates for each time point. To confirm the success of reprogramming, cells were analysed on the day 20 for markers of pluripotency: (1) cell morphology, (2) alkaline phosphatase expression, (3) pluripotent genes Nanog and Oct4 expression (Supplementary Fig. S1).

MEFs, partial iPS and fully reprogrammed iPS derived from EOS3F-24 line for corroborative experiment were a gift of Professor A. Hotta and were maintained as previously described^18^.

### Candidate genes and primer design

Table 1 contains information about the 12 commonly used hous™ekeeping genes chosen for this study, as well as 3 target genes (Nanog, Rex1, and Oct4) used for further validation, and the selected primer pairs used for amplification. All primers were designed according to MIQE guidelines^19^ with the aid of Primer-BLAST software (NCBI). Primers were designed to be specific preferentially for the longest isoform (transcript variant), allowing complementarity to other transcripts of the same gene (e.g. transcript variant 2, transcript variant X1 etc.), within the coding sequence. To select the best primers, the coding region of a gene was divided into portions spanning approximately 200–400 bp, and primers were designed to each portion using NCBI software. After excluding primer pairs that, according to NCBI Blast, could produce unintended target amplicons, resulting primer-pairs were tested by qPCR and the best pair for each gene was selected for the experiment.

**Table 1:** Summary of twelve housekeeping genes and three target genes (marked with asterisks*) evaluated in this study.

Gene symbol	Accession No.	Official Full Name (MGI)	Primer Pair (5′-3′)	size (bp)	Reference
Actb	NM_007393.5	actin, beta	TCGAGTCGCGTCCACC GGGAGCATCGTCGCCC	157	^22,35,38–44^
Atp5f1	NM_009725.4	ATP synthase, H + transporting, mitochondrial F0 complex, subunit B1	GTCCAGGGGTATTACAGGCAA TCAGGAATCAGCCCAAGACG	112	^44^
B2m	NM_009735.3	beta-2 microglobulin	ACGTAACACAGTTCCACCCG CAGTCTCAGTGGGGGTGAAT	150	^22,38,41,42^
Gapdh	NM_001289726.1	glyceraldehyde-3-phosphate dehydrogenase	GCACAGTCAAGGCCGAGAAT GCCTTCTCCATGGTGGTGAA	151	^35,38,40–44^
Gusb	NM_010368.2	glucuronidase, beta	AACAACACACTGACCCCTCA ACCACAGATCGATGCAGTCC	140	^41,42^
Hprt	NM_013556.2	hypoxanthine guanine phosphoribosyl transferase	CAGTCCCAGCGTCGTGATTA TGGCCTCCCATCTCCTTCAT	168	^22,35,38,42,43^
Pgk1	NM_008828.3	phosphoglycerate kinase 1	GGGTGGATGCTCTCAGCAAT GTTCCTGGTGCCACATCTCA	160	^38,44^
Ppia	NM_008907.1	peptidylprolyl isomerase A	CCAGTGCTCAGAGCTCGAAA	116	^15^
Rps18	NM_011296.2	ribosomal protein S18	CTAGACCGTTGGCCAGAACC	171	^22,45^
Tbp	NM_013684.3	TATA box binding protein	GCTACTGAACTGCTGGTGGG	160	^22,41,42^
Tfrc	NM_001357298.1	transferrin receptor	ACGGTCTGGTTCCTCATAACC	190	^45^
Ywhaz	NM_011740.3	tyrosine 3-monooxygenase/tryptophan 5-monooxygenase activation protein, zeta polypeptide	CCTTCTGCACCAGCTCATTT	190	^22,38,41^
Nanog*	NM_028016.3	Nanog homeobox	ATGCGTTCACCAGATAGCCC	122	
Rex1*	NM_009556.3	zinc finger protein 42	TGTTGACTACTGCCAAAGTTGGCC	174	
Oct4*	NM_013633.3	POU domain, class 5, transcription factor 1	CAGCAGATCACTCACATCGC GGGGCAGAGGAAAGGATACAG	175	

Accession numbers, gene descriptions, primer sequences and product sizes are shown. References point to previous works that used these genes in qPCR studies^38–45^.

### RNA isolation and cDNA synthesis

Total RNA was extracted with RNeasy kit (Cat# 74106, Qiagen, Japan) from each biological sample according to the manufacturer’s instructions (on-column genomic DNA digestion was performed as per said instructions), and RNA concentration and absorbance ratios (A_260/280_ and A_260/230_) were measured by spectrophotometer Nanodrop 2000 Spectrophotometer (NanoDrop Technologies, Japan). Only the samples with A_260/280_ and A_260/230_ were used for further analysis. 300 ng of RNA from each sample was reverse-transcribed using Omniscript RT Kit (Cat# 205111, Qiagen) in a total volume of 20 µl to produce DNA that was subsequently assessed by spectrophotometric analysis and diluted to 100 ng/µl. Then, individual master mixes with each of the DNA-primer combination (e.g. ‘Day 0 - Atp5f1’, ‘Day 0 - B2m’ etc.) were created for 4 technical replicas, and the mixtures were distributed onto the qPCR plate (8 μl per reaction well).

The reprogramming process (day 0 - day 20) was repeated 3 times, thus 3 biological replicates were obtained for each time point.

### Quantitative real-time PCR

qPCR was performed using a CFX96 Connect apparatus (BioRad, Japan). The reactions were carried out in triplicate using intercalating dye SYBR Green-based PCR super-mix (BioRad), following the manufacturer’s instructions. Each reaction was performed in the final volume of 8 μL, primers were used at the concentration of 300 nM. Thermocycler program consisted of an initial hot start cycle at 95 °C for 3 min, followed by 32 cycles at 95 °C for 10 sec and 59 °C for 30 sec. To confirm product specificity, melting curve analysis was performed after each amplification (Supplementary Fig. S2).

### Immunostaining

For immunofluorescence analyses cells were grown on glass bottom 30-mm dishes coated with collagen type I (IWAKI #4970-011). On the day of immunostaining cells were briefly washed with PBS, fixed with 4% PFA (Santa Cruz #sc-281692) for 15 min at room temperature and permeabilized with 0.5% Triton in PBS with 10% FBS addition for 30 min. Primary antibodies were applied: Anti-Oct4 (Santa Cruz #sc-5279, 1/250 dilution), Anti-Nanog (Abcam #ab80892, 1/250 dilution), in PBS with 10% FBS addition, for 1 hour in room temperature. After washing cells were incubated with secondary antibodies: Anti-mouse Alexa Fluor® 594 (Cell Signaling #8890, 1/500 dilution) and Anti-rabbit Alexa Fluor® 488 (Cell Signaling #4412, 1/500 dilution) in PBS with 10% FBS addition for 1 hour in room temperature, then cells were washed 4 times with PBS and 2 mL PBS per dish was added for imaging.

### Alkaline phosphatase staining and imaging

For alkaline phosphatase staining cells were briefly washed with PBS, fixed for 5 min with 4% PFA (Santa Cruz #sc-281692) at room temperature and stained with Alkaline phosphatase kit II (Stemgent, #00-0055) according to the manufacturer’s protocol. Imaging was carried out on Olympus CKX41 inverted microscope.

### Statistical analyses

The assay performance evaluation was carried out as described in MIQE guidelines. Reaction efficiency E was calculated as E = 10^(1/slope) − 1 × 100^ and precision was calculated as the average of all standard deviation values across samples for each gene. Linear dynamic range (LDR) is defined as the highest to the lowest quantifiable copy number established by means of a calibration curve^19^, and covers at least 3 orders of magnitude, as advised. The interval at which the main experiments were carried out fell into the linear portion of the calibration curve. The linearity was determined by means of correlation coefficients (R^2^). Precision refers to intraassay variation^19^ and is expressed as standard deviation (SD) of technical replicates, as advised. For BestKeeper^20^ analysis, Ct values were input directly, and geometric and arithmetic mean as well as standard deviation and coefficient of variance were calculated by the program, according to which genes were subsequently ranked from most stable to least stable. For NormFinder^21^ analysis, Ct values were transformed to linear scale and the normalization factor was calculated as the geometric mean of candidate reference genes included in the dataset. GeNorm software analysis was performed by calculating the expression stability measure as defined in the geNorm paper^22^, pairwise variation was determined and genes were ranked according to their positions. RefFinder algorithm was used to produce comprehensive ranking. This algorithm integrates four major programs (geNorm, Normfinder, BestKeeper, and the Delta Ct method method^23^) to assign a weight value to an individual gene and calculates the geometric mean of the weights for the overall final ranking^24^. Time-course plots of the gene expression through the reprogramming process were performed using the JMP software (JMP®, Version v11, SAS Institute Inc., Cary, US). Variance analyses between time points were performed using ANOVA test followed by a post-hoc Tukey HSD test at *p* < 0.05.

### Data availability

Raw data of qPCR are made available on request.

## Results

### Assay performance evaluation

To evaluate the performance of the qPCR assay, we first generated calibration curves using tenfold serial dilutions and assessed the PCR efficiency denoted *E* (see Materials and Methods), linear dynamic range (LDR) and precision, as described in MIQE guidelines^19^. Results are shown in Table 2 and Supplementary Fig. S3. The mean amplification efficiency values ranged from 95% (Actb) to 163% (Gusb), corresponding to slopes of −3.45 and −2.39, respectively. Nine genes out of twelve fell within “good” range of PCR efficiency defined as 90% < *E < *110%, while Tfrc, Hprt and Gusb produced 114%, 143% and 163%, respectively. To ensure that this result was due to the gene behavior rather than primer design, we evaluated 4 primer pairs for each of these genes, designed to cover different regions of the genes, as well as tested these pairs both in the parental cell line before reprogramming (N31 cells) and after the reprogramming (iPS cells). The results for these primer pairs can be found in Supplementary Table S1.

**Table 2:** Assay performance characteristics showing PCR efficiency *E*, linear dynamic range (LDR), slope, precision and associated correlation coefficient R^2^ (see Materials and Methods).

Gene	E (%)	Slope	LDR (ng)	Precision	R^2^
Actb	95	−3.45	0.07–700	0.18	0.999
Atp5f1	104	−3.23	0.07–70	0.31	0.999
B2m	100	−3.31	0.07–70	0.22	0.999
Gapdh	96	−3.42	0.07–70	0.29	0.998
Gusb	163	−2.39	7–700	0.38	0.991
Hprt	143	−2.60	7–700	0.12	0.995
Pgk1	101	−3.29	0.07–700	0.26	1.0
Ppia	100	−3.32	0.07–70	0.15	0.997
Rps18	99	−3.34	0.07–700	0.16	0.998
Tbp	106	−3.18	0.7–700	0.21	0.997
Tfrc	114	−3.02	0.07–700	0.36	0.999
Ywhaz	102	−3.27	0.7–700	0.17	0.999

The LDR values were lowest for Hprt and Gusb and were in the range of 7–700 ng of template. Correlation coefficients, on the other hand, varied from 0.991 (Gusb) to 1.0 (Pgk1) and fell within acceptable range for all genes as all of them were > 0.99. Precision values ranged from 0.12 (Hprt) to 0.38 (Gusb). Overall these results show good performance of the qPCR assay except for Gusb and Hprt that performed less well in the lower concentration ranges (less than 7 ng).

### Expression variability of candidate reference genes during the iPS reprogramming process

**Figure 1:**
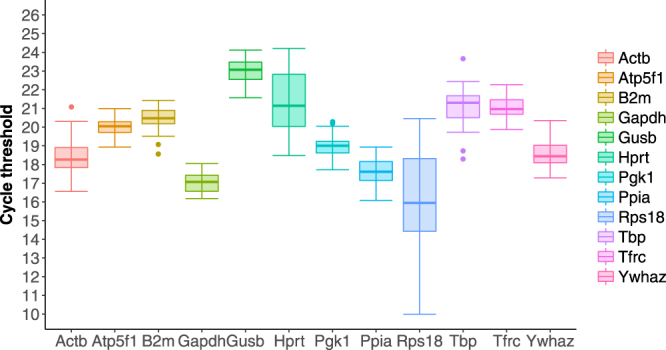
Box-and-whisker plot indicating range of Ct values of candidate reference genes throughout iPS reprogramming. Values of three biological replicates taken as averages of 4 technical replicates are given. The whiskers represent standard deviation of n samples (n = 24).

To assess the expression variability of chosen candidate reference genes during the reprogramming process, qPCR was performed and the relative Ct values for each gene across 8 time points were obtained throughout the reprogramming process, from day 0 to day 20. Figure 1 shows that the mean Ct values for 12 candidate genes varied from 9.99 to 24.21 cycles. The highest Ct value was observed for Rps18 (9.99) while the lowest value was observed for Hprt (24.21). To provide the initial estimation of the variability for each gene, we calculated the standard deviation and the coefficient of variation (CV). The least variable gene as expressed by SD value was Atp5f1 (*n* = 3, SD = 0.52), and the most variable was Rps18 (*n* = 3, SD = 2.70). Atp5f1 exhibited the lowest CV value, and Actb the highest one. For a more comprehensive analysis, the difference between 25^th^ and 75^th^ percentile was calculated in order to estimate the amplitude fluctuation. According to this analysis, Rps18 showed the highest variability, with an amplitude fluctuation of 3.89, and the least variable genes were Atp5f1 and Pgk1 with the amplitudes 0.58 and 0.61, respectively. Thus, according to the initial analysis, Atp5f1 was identified as the most stable gene while Rps18 was identified as the least stable gene across the whole reprogramming process.

### Time-course expression profiles of candidate reference genes

**Figure 2:**
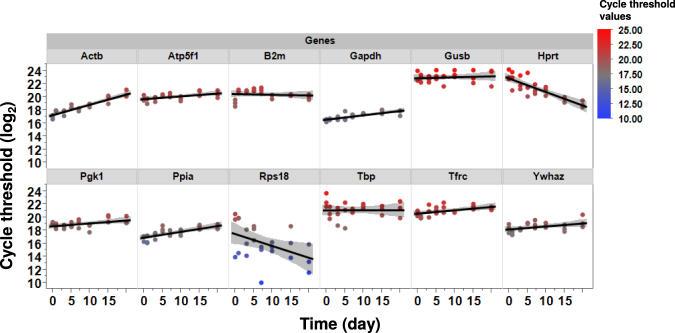
Expression profile of the 12 candidate reference genes throughout the 20 days of the reprogramming process. Measurements were performed in triplicate for each day. For each gene, linear fits were applied (black lines) and the displayed grey areas represent the 95% confidence intervals. For visualization purpose, we added a color bar representing the log2 values of Cycle threshold.

Figure 2 shows Cycle threshold values (Ct) of candidate reference genes plotted against time. The cycle threshold is inversely proportional to the gene expression of the considered gene. Results showed that Actb, Hprt, and Rps18 displayed the strongest variation over time (Fig. 2). According to this analysis, Actb expression decreased during the reprogramming process (R^2^ = 0.85), and the analysis of variance was found significant (*p* < 0.0001). Hprt and Rps18 expression decreased in the first week of reprogramming, increased around day 10, then decreased again. Rather than a linear fit, the values of Ct followed a cubic function. R^2^ values for Hprt and Rps18 were R^2^ = 0.71 and R^2^ = 0.20, respectively. At Day 20, the genes Gusb, Rps18, Tbp, and Ywhaz showed the strongest variations (Fig. 2).

### Analysis of candidate reference genes stability in iPS reprogramming process

The stability of candidate reference genes was analyzed according to four statistical methods of assessment, namely, the Delta Ct method, the estimation of the intra- and intergroup variation (NormFinder), the basic descriptive statistics evaluation (BestKeeper), and pairwise comparison (geNorm). The comprehensive ranking of the genes was also evaluated (see materials and methods), giving a total of five evaluation methods. The analysis revealed that Atp5f1 was unanimously chosen as the most stably expressed gene by all four algorithms, while Rps18 was designated as the least stable gene. Pgk1 was chosen as the second most stable gene by 4 out of 5 algorithms. Gapdh was designated as the third most stable gene, except by the Delta Ct method which designated it as the second most stable gene. Thus, the order of stability for the best three genes was summarized as follows: Atp5f1 >Pgk1 >Gapdh. On the other hand, the three least stable genes were Rps18 >Hprt >Tbp/Actb (Table 3).

**Table 3:** Ranking of the candidate reference genes according to five different evaluation methods.

Gene	Comprehensive Ranking		Delta Ct		geNorm		NormFinder		BestKeeper	
	Value	Rank	SD aver.	Rank	M value	Rank	Stability	Rank	SD	Rank
Atp5f1	1.00	1	1.03	1	0.362	1	0.288	1	0.40	1
Pgk1	1.86	2	1.12	3	0.362	1	0.489	2	0.46	2
Gapdh	2.91	3	1.11	2	0.504	2	0.516	4	0.48	3
Tfrc	4.23	4	1.14	4	0.560	3	0.640	5	0.52	4
Ppia	6.12	7	1.18	5	0.580	4	0.742	7	0.67	8
Gusb	5.66	5	1.22	7	0.729	6	0.502	3	0.60	7
Ywhaz	6.00	6	1.21	6	0.643	5	0.716	6	0.55	6
B2m	7.11	8	1.31	8	0.790	7	0.784	8	0.54	5
Actb	9.24	9	1.45	9	0.847	8	1.140	9	0.89	10
Tbp	9.74	10	1.59	10	0.952	9	1.194	10	0.86	9
Hprt	11.00	11	2.07	11	1.154	10	1.767	11	1.35	11
Rps18	12.00	12	2.88	12	1.441	11	2.750	12	2.11	12

Atp5f1, Pgk1 and Gapdh were ranked as the most stable candidate reference genes, while Rps18, Hprt, and Tbp/Actb were designated as the least stable ones.

Then, to corroborate our results in a more commonly used cell line, we assessed the stability of the 12 housekeeping genes using mouse embryonic fibroblasts (MEFs) described by Hotta *et al*.^18^. The stability was assessed using 3 time points for each gene, corresponding to the non-reprogrammed state (MEFs), partially reprogrammed state (partial iPS), and fully reprogrammed state (iPS). In fibroblasts, the five statistical algorithms also selected Atp5f1, Pgk1 and Gapdh as the best reference genes, while B2m, Actb and Hprt showed the lowest stability. (Supplementary Table S2 and Supplementary Fig. S4).

### Demonstration of the impact of the reference gene of choice on normalized values of the target genes

**Figure 3:**
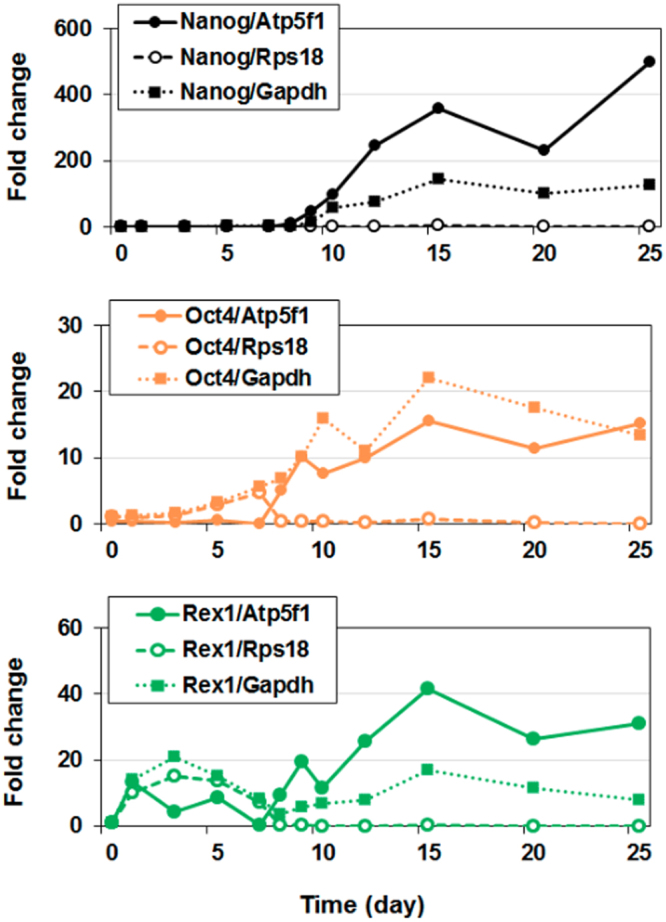
The impact of the choice of different reference genes on the target gene expression of three pluripotent markers. Fold change values of three pluripotency markers, Nanog, Oct4 and Rex1, were calculated using Atp5f1 (full line, filled circles) Rps18 (dashed line, white circles) and Gapdh (dotted line, filled squares). When using Atp5f1 as a reference gene, which was selected as the best candidate housekeeping gene, the fold change values greatly increased over time, corresponding to an increase in the expression of Nanog, Oct4 and Rex1. When using Rps18 as a reference gene, which was selected as the least stable gene during reprogramming, very low values of gene expression were obtained, without any increase during the later stages of reprogramming. Gapdh, a candidate reference gene ranked 2nd, 3rd or 4th among all tested genes, produced an increase in expression patterns of pluripotency genes, however, to a lower extent than the gene Atp5f1.

To assess the performance of chosen reference genes we used three common pluripotency markers, Nanog, Oct4 and Rex1, whose levels are known to increase during iPS reprogramming^25^ and normalized them against three different reference genes. From the results of above analyses and ranking (Table 3), we selected the worst candidate (Rps18), the best candidate (Atp5f1), and Gapdh which was assigned somewhat intermediate position among the candidates. Gapdh is often used as a reference genes in other studies. Figure 3 shows the fold change in gene expression of the pluripotent markers when normalized against these three genes using the classical Delta Delta Ct method in which day 0 of the reprogramming is set as the control (value 1). Results clearly demonstrate that the normalization against Atp5f1 caused the gene expression value to increase greatly during reprogramming (more than 400 fold for Nanog, more than 15 fold for Oct4, and more than 35 fold for Rex1), as it would be expected from the biological perspective. The normalization against Rps18, the worst candidate reference, does not show such increase and demonstrates very low pluripotency genes values towards the end of the reprogramming. Normalization to Gapdh caused the gene expression of the pluripotency genes to increase, although some fluctuations were observed, and the gene expression values at the end of reprogramming were significantly lower for Nanog (under 200 fold) and Rex1 (under 20 fold), and higher for Oct4 (more than 20 fold).

## Discussion

iPS reprogramming represents a forced change in cell fate and is associated with profound alterations in gene expression. In particular, it is known that these alterations include most basic, “housekeeping” functions such as cell metabolism^4^, speed of the cell cycle^9^ and lipid profile^26,27^. When normalizing qPCR data to a reference gene, it is of crucial importance to make sure that the gene of choice is stably expressed throughout all experimental conditions. The need to confirm stability of expression is even greater in case of iPS reprogramming, as housekeeping genes could be affected by dramatic changes in chemical metabolism of the cells. In this work, we performed iPS reprogramming in murine cells and measured the expression patterns of the most commonly used housekeeping genes. To assess the suitability of these genes as reference for qPCR experiments, we analyzed the expression data based on five statistical algorithms previously reported^20–22^.

Based on our statistical analyses, we found that out of 12 selected genes, the glycolytic enzymes Pgk1 and Gapdh, as well as the mitochondrial Atp5f1, have outperformed other genes for stability on the mRNA level. (Fig. 1 and Supplementary Fig. 4). First of all, the Atp5f1 gene, which had the highest stability rank, is a B subunit of the proton channel of mitochondrial F0 complex, and is a part of mitochondrial ATP synthase. ATP synthase is composed of F0 and F1 complexes and is linked by the peripheral stalk, of which B subunits are part^28^. The function of the subunits in this context is, apart from linking the complexes, to act as a stator to prevent other subunits from rotation in relation to the central rotary element. Atp5f1 is, thus, an essential structural element of the ATP synthase. The second most stable gene as determined by four algorithms was phosphoglycerate kinase 1 (Pgk1). It is an ATP-generating enzyme that catalyzes the reversible conversion of 1,3-diphosphoglycerate and ADP to 3-phosphoglycerate and ATP and is considered an important part of the glycolytic pathway^29^. The third most stable gene was Gapdh, or glyceraldehyde 3-phosphate dehydrogenase, which also belongs to the glycolytic pathway, and catalyses the conversion of glyceraldehyde 3-phosphate to D-glycerate 1,3-bisphosphate. These results can be explained from the point of view of requirement for glycolysis. Recent research has shown that glycolysis is required for iPS reprogramming^30^ and that inhibition of glycolysis can impede the reprogramming process^31^. Our data suggest that such glycolytic proteins as Pgk1 and Gapdh, or a part of ATP synthase Atp5f1 may provide the most stable reference for mRNA analysis during iPS reprogramming.

On the other hand, our analysis has revealed that the expression pattern of the actin gene (Actb), often considered as a reliable reference gene in other studies^32^, steadily decreased throughout the reprogramming. As a result, this gene was consistently ranked as one of the most unstable genes across different statistical methods. This change in actin mRNA expression may reflect the cytoskeletal remodeling which is normally associated with iPS reprogramming^33^ and plays a central role in the cell fate change^34^. Thus, in our opinion, when doing experiments related to cell stemness, actin should be regarded as a target gene whose expression is affected by the experiment rather than a stable reference gene.

Hprt was marked as second least stable gene in our analysis. This result is in agreement with previous work on reference genes in pluripotent stem cells that also marked Hprt unsuitable for use as a reference gene^35^. The authors conducted differentiation of embryonic stem cells and measured housekeeping gene expression change at different time points. Induction of pluripotency can be viewed as a process opposite to differentiation, with pluripotency features gradually emerging instead of disappearing. In that regard, it is important to note that Gapdh in the mentioned work was also found suitable for use as a reference gene, as it was in our analysis.

The ribosomal gene Rps18 has been found to vary greatly and stand out as the most unstable gene among all, being ranked last by all algorithms unanimously. Previous investigations of Rps18 have shown that this gene can be stable^36^ or unstable^37^ as a reference for qPCR experiments. Our study has found that, in addition to high variability, the expression level of Rps18 was very high compared to the majority of other genes (the average of 16.09 cycles, compared to other genes having around 20 cycles on average), and it increased during reprogramming. This level and the increase may reflect the growing need of the cell in protein synthesis because of metabolic alterations and increased proliferation rate. Such large differences in basal expression and high variability make Rps18 an unsuitable candidate for normalization, and its use as a reference should be avoided in future works on iPS reprogramming.

As a case in point, we performed qPCR for three pluripotency markers Nanog, Oct4 and Rex1 (Fig. 3) and demonstrated that the choice of reference genes is determinant to accurately describe the gene expression of target genes throughout the reprogramming process. It is unfortunate that most studies do not provide substantial evidence to support their choice for the housekeeping gene used in calculation of gene expression. To our knowledge, this is the first report to provide a detailed investigation of the potential candidate reference genes in mammalian cells during iPS reprogramming. We hope that this study will benefit further studies, and encourage the community to perform systematic investigation of suitable reference genes prior to gene expression analyses.

## Electronic supplementary material

Supplementary Information
