# Supplementary material for: Validation of Common Housekeeping Genes as Reference for qPCR Gene Expression Analysis During iPS Reprogramming Process
Source: Sci Rep. 2018 Jun 7;8:8716. doi: 10.1038/s41598-018-26707-8 (PMC5992140; doi:10.1038/s41598-018-26707-8)
Supplement: Supplementary file 1 — Supplementary Information [file 41598_2018_26707_MOESM1_ESM.pdf]

Validation of Common Housekeeping Genes as Reference for qPCR Gene Expression Analysis During iPS Reprogramming Process

Yulia Panina[1,2] , Arno Germond[1] , Shinji Masui[3] , Tomonobu M. Watanabe*[1,2]
Laboratory for Comprehensive Bioimaging, RIKEN QBiC, Osaka, Japan

[1] RIKEN Quantitative Biology Center (QBiC), 6-2-3 Furuedai, Suita, Osaka 565-0874, Japan
[2] Graduate School of Frontier Biosciences, Osaka University, 1-3 Yamadaoka, Suita, Osaka 565-0871, Japan
[3] Department of Life Science Frontiers, Center for iPS Cell Research and Application (CiRA), Kyoto University, 53 Kawahara-cho, Shogoin, Sakyo-ku, Kyoto 606-8507, Japan
*Corresponding author: Tomonobu M. Watanabe, tomowatanabe@riken.jp

Supplementary Tables

Cell type	Gene	Pair #	Primer sequence	Slope	E (%)	R^2
Fully reprogrammed iPS cells	Gusb	1	F: AACAACACACTGACCCCTCA<br>R: ACCACAGATCGATGCAGTCC	1.054	93	0.953
	Gusb	2	F: TGGCTGGGTGTGGTATGAAC<br>R: GGTGACCTCCCTCATGTTCC	0.722	161	0.931
	Gusb	3	F: GGTGGAACATGAGGGAGGTC<br>R: AGGGTATGAGGGGTCAGTGT	0.926	111	0.983
	Gusb	4	F: GGCCTCTAGATAGCCTTGAGC<br>R: ACACGCACTCCATTTTAGGGA	0.342	660	0.736
	Hprt	1	F: GTTGGGCTTACCTCACTGCT<br>R: TAATCACGACGCTGGGACTG	0.526	274	0.923
	Hprt	2	F: GATCAGTCAACGGGGGACAT<br>R: GGTCCTTTTCACCAGCAAGC	0.529	271	0.886
	Hprt	3	F: ACAGGCCAGACTTTGTTGGA<br>R: ACTTGCGCTCATCTTAGGCT	0.699	170	0.962
	Hprt	4	F: CAGTCCCAGCGTCGTGATTA<br>R: TGGCCTCCCATCTCCTTCAT	1.01	99	0.995
	Tfrc	1	F: AAACTGGCTGAAACGGAGGA<br>R: AGATCCAGCCTCACGAGGAG	1.157	82	0.997

	Tfrc	2	F: AAGAGCTGCTGCAGAAAAGC<br>R: ACGGTCTGGTTCCTCATAACC	1.049	94	0.997
	Tfrc	3	F: GTTCGTACAGCAGCGGAAGT<br>R: GGAAGTAGTCTCCACGAGCG	1.073	91	0.985
	Tfrc	4	F: AGCAAAGTCTGGCGAGATGAA<br>R: CCACATAACCCTCGGGAGAC	1.189	79	0.989
Parental cells (N31)	Gusb	1	F: AACAACACACTGACCCCTCA<br>R: ACCACAGATCGATGCAGTCC	-2.39	163	0.991
	Gusb	2	F: TGGCTGGGTGTGGTATGAAC<br>R: GGTGACCTCCCTCATGTTCC	-1.69	289	0.988
	Gusb	3	F: GGTGGAACATGAGGGAGGTC<br>R: AGGGTATGAGGGGTCAGTGT	-2.32	170	0.980
	Gusb	4	F: GGCCTCTAGATAGCCTTGAGC<br>R: ACACGCACTCCATTTTAGGGA	-1.30	486	0.955
	Hprt	1	F: GTTGGGCTTACCTCACTGCT<br>R: TAATCACGACGCTGGGACTG	-2.23	181	0.979
	Hprt	2	F: GATCAGTCAACGGGGGACAT<br>R: GGTCCTTTTCACCAGCAAGC	-1.49	367	0.976
	Hprt	3	F: ACAGGCCAGACTTTGTTGGA<br>R: ACTTGCGCTCATCTTAGGCT	-1.40	423	0.960
	Hprt	4	F: CAGTCCCAGCGTCGTGATTA<br>R: TGGCCTCCCATCTCCTTCAT	-2.60	143	0.995
	Tfrc	1	F: AAACTGGCTGAAACGGAGGA<br>R: AGATCCAGCCTCACGAGGAG	-3.16	107	0.987
	Tfrc	2	F: AAGAGCTGCTGCAGAAAAGC<br>R: ACGGTCTGGTTCCTCATAACC	-3.02	114	0.999
	Tfrc	3	F: GTTCGTACAGCAGCGGAAGT<br>R: GGAAGTAGTCTCCACGAGCG	-3.08	111	0.972
	Tfrc	4	F: AGCAAAGTCTGGCGAGATGAA<br>R: CCACATAACCCTCGGGAGAC	-3.28	102	0.991

Supplementary Table S1. Assay performance characteristics on 4 different primer pairs for Gusb, Hprt and Tfrc evaluated in parental cell line and in fully reprogrammed iPS cells. PCR efficiency *E*, slope, and associated correlation coefficient R^2 are shown. The serial dilutions

in fully reprogrammed iPS cells were twofold.The efficiency for twofold dilutions was calculated using the formula E=2^(1/slope)-1*100. The serial dilutions in parental cells were tenfold, and the formula for calculating efficiency was E=10^(1/slope)-1*100. In fully reprogrammed iPS cells, the primer pair closest to 100% efficiency was #1 for Gusb (E=93%), #4 for Hprt (E=99%), and #2 for Tfrc (E=94%). The efficiency for the same sets of primers differed in parental cells, giving E=163% for Gusb, E=143% for Hprt, and E=114% for Tfrc. The best pair of primers for each gene (Gusb #1, Hprt #4 and Tfrc #2) was chosen for the main experiment.

Gene	Comprehensive Ranking		Delta Ct		geNorm		NormFinder		BestKeeper	
	Value	Rank	SD aver.	Rank	M value	Rank	Stability	Rank	SD	Rank
Atp5f1	1.57	1	0.49	1	0.333	5	0.099	1	0.077	1
Pgk1	1.68	2	0.50	2	0.168	1	0.251	2	0.247	2
Gapdh	3.44	3	0.55	5	0.168	1	0.417	7	0.322	4
Ppia	3.66	4	0.56	3	0.197	2	0.353	4	0.323	5
Gusb	4.53	5	0.59	4	0.300	4	0.311	3	0.345	7
Tbp	5.18	6	0.60	6	0.467	7	0.400	5	0.285	3
Tfrc	6.88	7	0.62	7	0.253	3	0.458	8	0.422	10
Ywhaz	7.20	8	0.64	8	0.415	6	0.414	6	0.367	8
Rps18	8.80	9	0.65	10	0.532	9	0.564	10	0.337	6
Hprt	9.00	10	0.70	9	0.501	8	0.464	9	0.395	9
Actb	11.00	11	0.74	11	0.560	10	0.631	11	0.429	11
B2m	12.00	12	1.03	12	0.638	11	0.961	12	0.879	12

Supplementary Table S2. Ranking of the candidate reference genes' stability during reprogramming according to five different evaluation methods in mouse embryonic fibroblasts (MEFs). Atp5f1, Pgk1 and Gapdh were ranked as the most stable candidate reference genes overall, while Hprt, Actb and B2m were designated as the least stable ones.

Supplementary figures

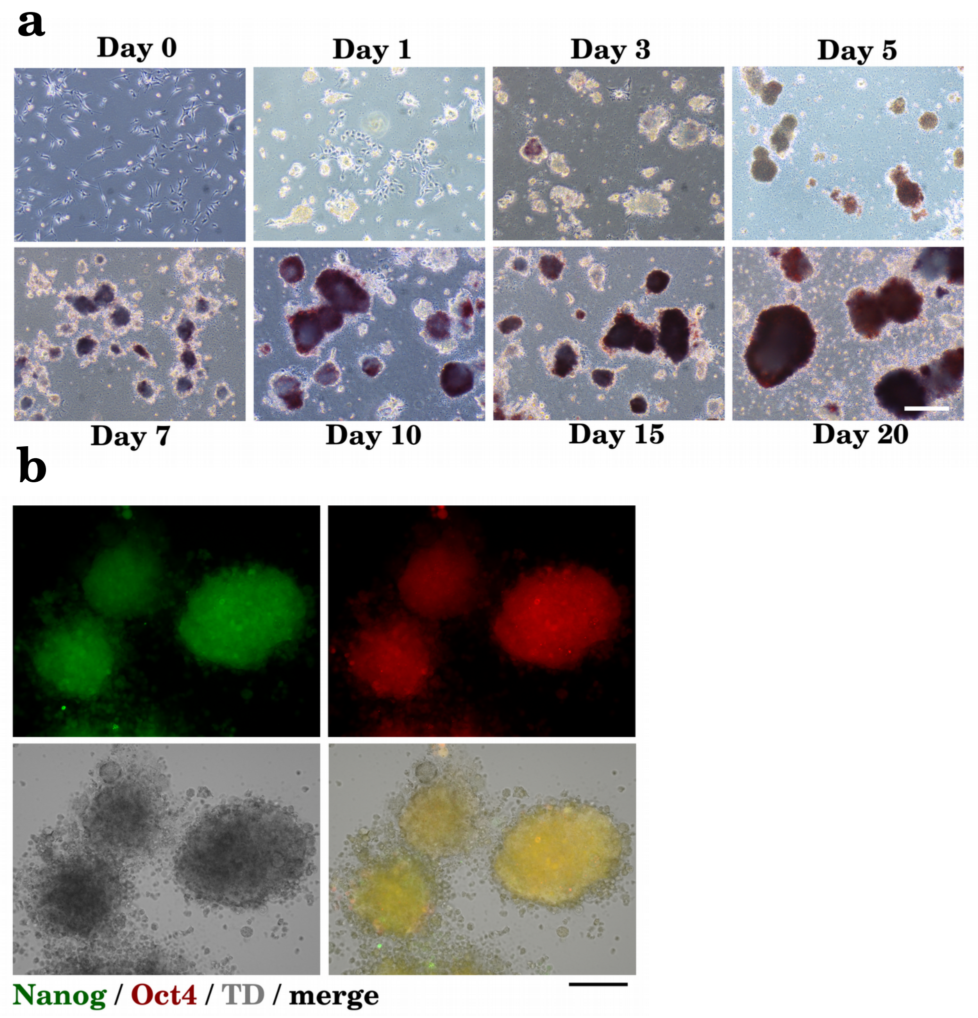

Supplementary Figure S1. Pluripotency markers check in the reprogramming process of N31 cell line. (a) Alkaline phosphatase staining of nascent iPS colonies over the course of iPS reprogramming. (b) Immunostaining of fully reprogrammed iPS colonies at day 20 with pluripotency markers anti-Nanog and anti-Oct4.

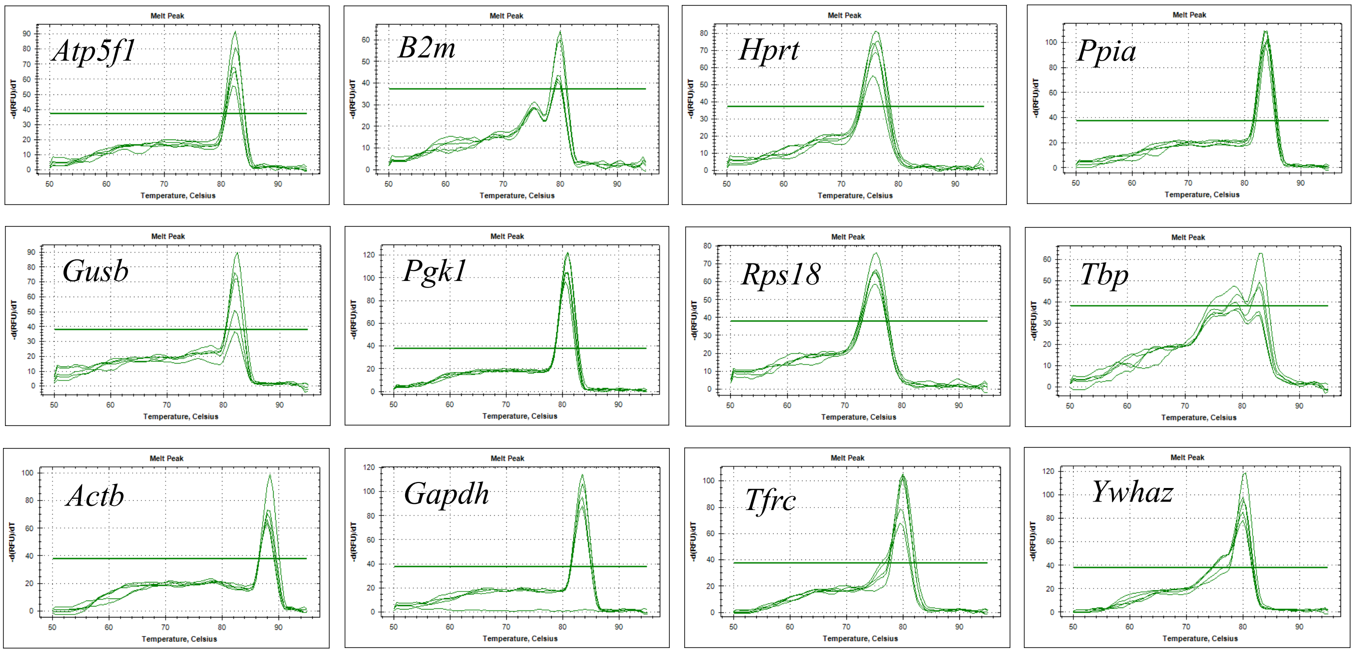

Supplementary Figure S2. Melting curves of candidate reference genes. Curves from log 10 dilution series are included for demonstration.

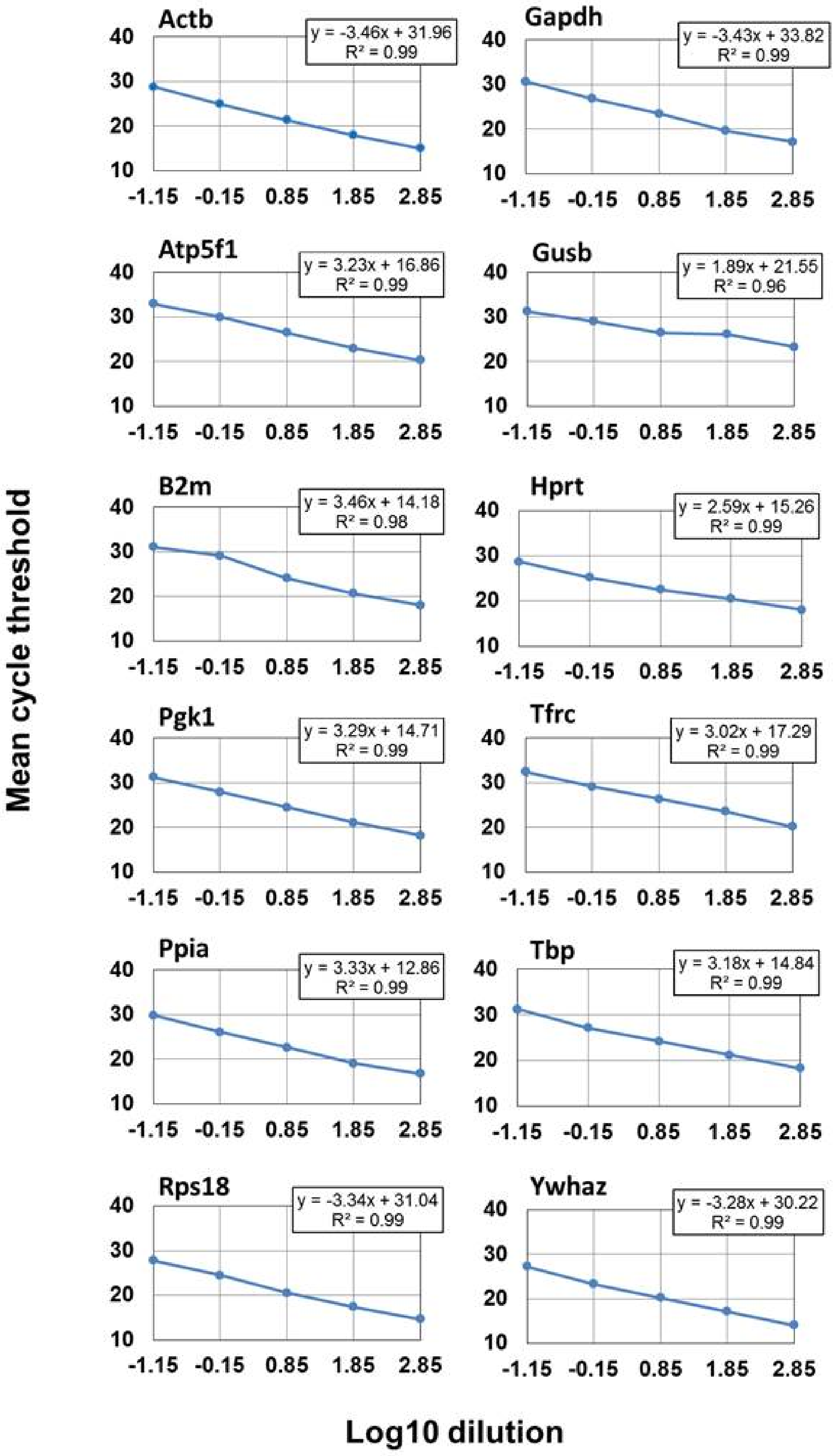

Supplementary Figure S3. Log 10 dilution curves of candidate reference genes. The log 10 dilution values were plotted against cycle threshold values for evaluation of linearity. Correlation coefficients are included for each curve.

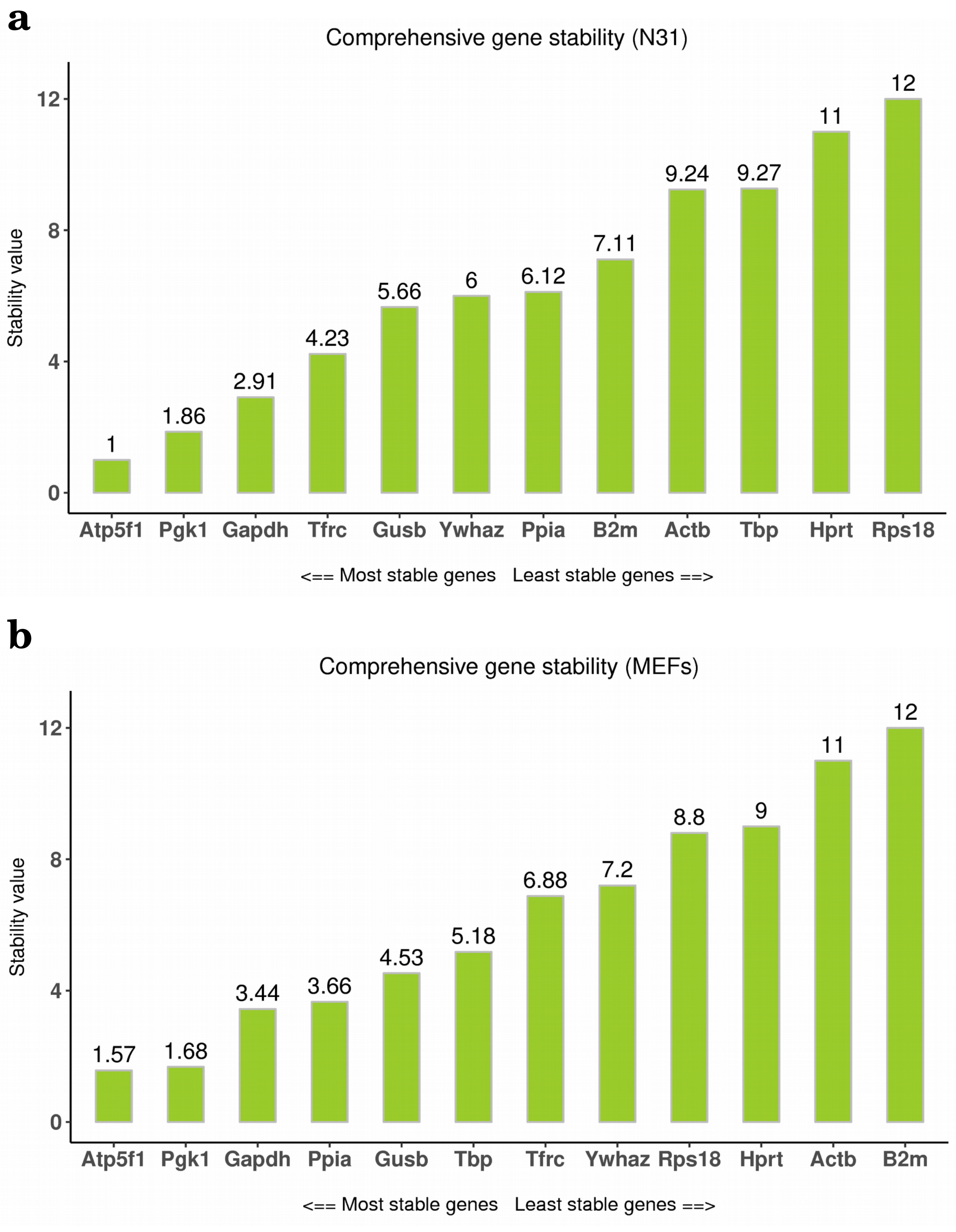

Supplementary Figure S4. Comprehensive stability ranking of candidate reference genes during iPS reprogramming of neural stem cells (N31) or mouse embryonic fibroblasts (MEFs). The stability is expressed as a comprehensive RefFinder value which is a geometric mean of the overall weight value for four algorithms: Genorm, Normfinder, Bestkeeper and Delta Ct (see Materials and Methods). Atp5, Pgk1 and Gapdh are showing the best stability in both cell lines. The lowest stability values differed in two cell lines, the lowest score belonging to Rps18, Hprt and Tbp in N31 cells (a) and to B2m, Actb and Hprt in MEFs (b).
